# Postoperative pneumatosis intestinalis (PI) and portal venous gas (PVG) may indicate bowel necrosis: a 52-case study

**DOI:** 10.1186/s12893-016-0158-x

**Published:** 2016-07-08

**Authors:** Kazuya Higashizono, Hideaki Yano, Ouki Miyake, Kunihiro Yamasawa, Masanori Hashimoto

**Affiliations:** Department of Surgery, National Center for Global Health and Medicine, 1-21-1 Toyama, Shinjuku-ku, Tokyo, 162-0054 Japan; 7-3-1 Hongo, Bunkyo-ku, Tokyo, 113-8655 Japan

**Keywords:** Bowel necrosis, Pneumatosis intestinalis, Hepatic portal venous gas

## Abstract

**Background:**

The significance of pneumatosis intestinalis (PI) and portal venous gas (PVG) is controversial. This retrospective study evaluated the risk factors for bowel necrosis in patients with PI and/or PVG.

**Methods:**

Between 2002 and 2015, 52 patients were diagnosed with PI and/or PVG and were included in this study. The patients were classified according to the presence or absence of bowel necrosis in surgical findings or at autopsy. Patient characteristics and clinical findings related to bowel necrosis were investigated.

**Results:**

Bowel necrosis was diagnosed in 17 (32.7 %) patients. Amongst these 17, 10 patients received salvage surgical intervention, and seven of those diagnosed with bowel necrosis survived after the operation. The remaining 35 patients received conservative treatment with or without exploratory laparotomy. Between patients with and without bowel necrosis, laboratory data revealed significant differences in the levels of C-reactive protein (*P* = 0.0038), creatinine (*P* = 0.0054), and lactate (*P* = 0.045); clinical findings showed differences in abdominal pain (*P* = 0.019) and peritoneal irritation signs (*P* = 0.016); computed tomography detected ascites (*P* = 0.011) and changes of bowel wall enhancement (*P* = 0.03) that were significantly higher in patients with bowel necrosis. The rate of PI and/or PVG detected in patients postoperatively was significantly higher in patients with bowel necrosis (*P* < 0.0001). Multivariate analysis showed that bowel necrosis was significantly more likely when PI or PVG was detected in postoperative patients than in patients who had not had surgery (*P* = 0.003).

**Conclusions:**

PI and/or PVG, alone, are not automatically indicative of bowel necrosis. However, when these conditions occur postoperatively, they indicate bowel necrosis requiring reoperation.

## Background

Pneumatosis intestinalis (PI) and the presence of hepatic portal venous gas (PVG) have long been thought to predict bowel necrosis and an associated poor prognosis [1–3]. However, widespread use of computed tomography (CT) has allowed better and more frequent detection of these conditions [4, 5]. PI and PVG are suggested to have various sequelae, including bowel necrosis, which are associated with poor patient outcomes. However, the diagnosis of bowel necrosis is often difficult in patients with PI and/or PVG. Accordingly, we investigated patients with PI/PVG to identify the predictors of bowel necrosis.

## Methods

### Study groups

Between January 2002 and January 2014, abdominal CT scan was used to diagnose PI/PVG in 57 patients at the National Center for Global Health and Medicine (Tokyo, Japan). Five patients were excluded from the study due to 1) disturbed consciousness or cardiopulmonary arrest upon arrival, 2) post-mortem CT, or 3) patient age < 18 years. The remaining 52 patients were classified as having or not having bowel necrosis. Bowel necrosis was diagnosed based on surgical findings and/or postoperative pathologic examinations or autopsies. The clinical characteristics, laboratory/imaging findings, and condition severity of the patients in the two groups were compared to identify the indicators of bowel necrosis.

### CT image interpretation

Study group information was extracted from a database containing interpretations of imaging studies performed by experienced radiologists, using the key words: “pneumatosis intestinalis (PI)” and “portal venous gas (PVG).” Gas within 2 cm of the liver border in the contrast-enhanced images was defined as PVG [1]. If a contrast-enhanced CT image showed a decrease in bowel wall enhancement, bowel ischemia was suspected.

### PVG evaluation and PI distribution

Patients with PVG were evaluated to determine whether the amount of gas was a predictor of outcome. PVG generally expands from the left lobe of the liver to the right anterior lobe and then to the right posterior lobe as the amount of gas increases [6]. Therefore, we compared patients with gas confined to the left lobe to those with gas in the right lobe or in both lobes. PI severity is reported to be related to its distribution [7], so patients with PI were classified according to whether they had a bubble type or linear gas pattern.

### Severity evaluation

Disease severity was evaluated using the following three scoring systems: (1) Acute Physiology and Chronic Health Evaluation II (APACHE II) score; (2) Sequential Organ Failure Assessment (SOFA) score; and (3) Systemic Inflammatory Response Syndrome (SIRS) score. These scores were calculated using clinical findings and laboratory parameters at the time PI/PVG was diagnosed.

### Statistical analysis

Statistical analyses were performed using JMP 10 (SAS Institute, Cary, NC, USA). Univariate analyses were performed using chi-square and Fisher’s exact tests; multivariate analyses were performed using logistic regression analysis. A probability (*P*) value < 0.05 indicated statistical significance.

## Results

### Patient characteristics

The 52 patients comprised 32 (61.5 %) men and 20 (38.5 %) women, with a mean age of 70.4 years (range, 19–97 years; median, 74 years) (Table 1). Their underlying diseases included diabetes, respiratory disease, oncologic disease, collagen disease, cardiovascular disease, renal disease, hematological disorder, and inflammatory bowel disease; 16 patients (30.7 %) were taking steroids. Of the included patients, 17 were diagnosed with bowel necrosis, and 35 patients were determined not to have bowel necrosis. In patients with bowel necrosis, diagnoses included ileus (*n* = 6), non-occlusive mesenteric ischemia (NOMI; *n* = 3), superior mesenteric artery thromboembolism (*n* = 2), bowel perforation (*n* = 2), ischemic enteritis (*n* = 2), fatal toxic megacolon (*n* = 1), and postoperative abscess (*n* = 1). In patients without necrosis, diagnoses ranged from infectious enteritis to simple obstruction; the cause of symptoms was unknown in 11 patients. Of the 17 patients with bowel necrosis, 7 (41.2 %) survived, but required surgical intervention (Table 1). Ten of the 17 patients died during their hospitalization. Of the 35 patients without necrosis, 2 died due to pneumonia. The incidence of postoperative PI/PVG detection was significantly higher in patients with bowel necrosis (*P* < 0.0001); none of the other patient characteristics was associated with the incidence of detection.

**Table 1:** Patient characteristics, clinical findings, laboratory and computed tomography findings

	Total	Bowel necrosis	Non-bowel necrosis	
Number (N, (%))	*N* = 52	*N* = 17 (32.7)	*N* = 35 (67.3)	*P* value
Age	70.4 ± 5.1	69.5 ± 8.9	70.9 ± 6.2	0.79
Gender				0.74
Male	32 (61.5)	11	21	
Female	20 (38.5)	6	14	
Underlying disease/condition/treatment				
Inflammatory bowel disease	2 (3.8)	0	2	0.31
Respiratory disease	7 (13.5)	2	5	0.8
Diabetes mellitus	10 (19.2)	3	7	0.84
α-Glucosidase inhibitor	5 (9.6)	1	4	0.52
Cardiovascular disease	9 (17.3)	4	5	0.41
Renal disease	4 (7.7)	2	2	0.44
Collagen disease	7 (13.5)	2	5	0.8
Hematological disease	3 (5.8)	0	3	0.21
Steroid therapy	16 (30.8)	4	12	0.43
Chemotherapy	8 (15.4)	3	5	0.75
Postoperative state	9 (17.3)	8	1	0.0001
Tumor-bearing	13 (25.0)	5	8	0.61
Abdominal findings				
Abdominal pain	31 (59.6)	14 (82.4)	17 (48.6)	0.019
Peritoneal irritation signs	16 (30.8)	9 (52.9)	7 (20.0)	0.016
Nausea/vomiting	17 (32.7)	7 (41.2)	10 (28.6)	0.36
Abdominal distension	26 (50)	11 (64.7)	15 (42.9)	0.14
Melena	5 (9.6)	1 (5.9)	4 (11.4)	0.52
Vital signs				
Temperature		36.9 ± 0.66	37.1 ± 0.5	0.76
Mean blood pressure		79.6 ± 8.9	86.9 ± 7.8	0.24
Pulse rate		94.2 ± 13.6	85.6 ± 11.6	0.34
Respiration rate		19.3 ± 2.6	17.9 ± 1.8	0.36
Laboratory Findings				
WBC (×10^3^/μL)		11,992 ± 2811	8654 ± 1988	0.16
Hb (g/dL)		12.3 ± 0.97	11.8 ± 0.68	0.39
Platelets (×10^3^/μL)		18.9 ± 4.1	19.9 ± 2.9	0.67
Total bilirubin (mg/dL)		1.16 ± 0.5	0.90 ± 0.35	0.39
Sodium (mmol/L)		135.2 ± 3.2	137.4 ± 2.3	0.26
Potassium (mmol/L)		4.36 ± 0.34	4.2 ± 0.2	0.49
Arterial pH		7.338 ± 0.065	7.409 ± 0.049	0.81
Base excess (mmol/L)		−0.66 ± 0.32	1.22 ± 0.38	0.41
Bicarbonate (mmol/L)		22.39 ± 3.62	24.92 ± 2.61	0.28
Creatinine (mg/dL)		1.7 ± 0.4	0.9 ± 0.3	0.0054
CRP (mg/dL)		10.56 ± 4.01	3.15 ± 2.83	0.0038
Serum lactate (mmol/L)		4.54 ± 2.03	1.90 ± 1.61	0.045
Evaluation of Severity				
SIRS score		1.1 ± 0.5	0.4 ± 0.3	0.036
APACHE II score		12 ± 2.9	7.7 ± 2.1	0.022
SOFA score		2.8 ± 0.9	1.2 ± 0.6	0.0044
Computed tomography findings				
Ascites	18 (34.6)	10 (58.8)	8 (22.9)	0.011
Free Air	20 (38.5)	5 (29.4)	15 (42.9)	0.35
Pneumatosis intestinalis (PI)	45 (86.5)	14 (82.3)	31 (88.6)	0.54
linear pattern	23 (51.1)	10 (58.8)	13 (37.1)	
bubble pattern	22 (48.9)	4 (23.5)	18 (51.4)	0.082
Portal venous gas (PVG)	20 (38.5)	9 (52.9)	11 (31.4)	0.13
Left lobe	12 (23.1)	5 (29.4)	7 (20)	
Right lobe or both lobes	8 (15.4)	4 (23.5)	4 (11.4)	0.71
Both PI and PVG	13 (25)	6 (35.3)	7 (20)	0.23
Deterioration in bowel wall enhancement	17 (32.7)	8 (47.1)	9 (25.7)	0.03

Bubble pattern: gas bubbles in the bowel wall

Linear pattern: continuous gas with a smooth margin

### Clinical, laboratory, and imaging findings

Clinical findings included abdominal pain, abdominal distension, nausea and vomiting, peritoneal irritation signs, and melena (Table 1). A significant difference was observed between the two groups with respect to abdominal pain (*P* = 0.019) and peritoneal irritation signs (*P* = 0.016). Conversely, there were no significant differences in vital signs. There were also significant differences in the levels of C-reactive protein (CRP) (*P* = 0.0038), creatinine (*P* = 0.0054), and lactate (*P* = 0.045) between the two groups (Table 1). In patients with bowel necrosis, levels of CRP (10.56 ± 4.01 mg/dL), creatinine (1.7 ± 0.4 mg/dL), and lactate (4.54 ± 2.03 mmol/L) were higher than the levels in patients without bowel necrosis. With respect to patient condition severity, statistically significant differences were found between patients with and without bowel necrosis in the APACHE II, SOFA, and SIRS scores. The APACHE II (12 ± 2.9), SOFA (2.8 ± 0.9), and SIRS (1.1 ± 0.5) scores were higher in patients with bowel necrosis. Among all patients, CT images revealed abdominal ascites in 18 (34.6 %) and free air in 20 (38.5 %) (Table 1); bowel wall enhancement changes were observed in 17 patients (32.7 %).

PI was detected in 45 patients (86.5 %), and PVG was found in 20 (38.5 %), with 13 patients (25 %) exhibiting both; the presence of PI and/or PVG was not significantly related to bowel ischemia, however. Among the 20 patients with PVG, gas was confined to the left lobe in 12 patients and was seen in the right or in both lobes in 8. No gas location was significantly associated with obowel ischemia (*P* = 0.71). Bowel necrosis was more frequent in patients with a linear gas pattern, but the difference was not significant (*P* = 0.082). However, changes in bowel wall enhancement were significantly more frequent in patients with bowel necrosis (*P* = 0.03).

### Treatment and outcomes

Surgery was performed on 11 (21.2 %) patients, and 41 (78.8 %) were treated conservatively (Table 2). Operations included right hemicolectomy with massive bowel resection (*n* = 4), alleviation of ileus and partial small bowel resection (*n* = 2), intraperitoneal irrigation and drainage (*n* = 2), Hartmann’s operation (*n* = 1), and exploratory laparotomy (*n* = 2). One patient who underwent exploratory laparotomy had a preoperative diagnosis of sigmoid colon necrosis, but the laparotomy did not reveal bowel necrosis. The other patient undergoing exploratory laparotomy had massive necrosis extending from the stomach to the large bowel, and definitive treatment was considered impossible. This patient died. Conservative treatment included bowel rest only (*n* = 13); observation plus antibiotic therapy (*n* = 17); decompression via a gastric/ileus tube (*n* = 6); oxygen therapy, including hyperbaric oxygen therapy (*n* = 3); and other treatments (*n* = 5). Bowel necrosis was suspected in 8 patients receiving conservative treatment, but laparotomies were not performed for various reasons. Seven of these 8 patients died from deterioration of their general condition due to bowel necrosis or the underlying disease. One patient, who probably had transient bowel ischemia, demonstrated an improved clinical state and achieved a good outcome.

**Table 2:** Operative method for PI/PVG patients

Treatment	Total	Bowel necrosis	Non-bowel necrosis	
	*N* = 52	*N* = 17 (32.7)	*N* = 35 (67.3)	*P* value
∙ Surgery	11 (21.2)	10 (58.8)	1 (2.9)	
Right hemicolectomy ± massive bowel resection	4	4	0	
Ileus surgery ± partial small bowel resection	2	2	0	
Hartmann’s operation	1	1	0	
Intraperitoneal irrigation and drainage	2	2	0	
Exploratory laparotomy	2	1	1	
∙ Conservative therapy	41 (78.8)	7 (41.2)	33 (97.1)	
No oral treatment	13	0	13	
No oral treatment + Antibiotic	17	5	12	
Decompression via gastric tube	6	2	4	
Oxygen therapy	3	0	3	
Other	5	0	5	
In-hospital deaths	12 (23.1)	10 (58.8)	2 (5.7)	<0.0001

### Univariate/multivariate analyses

In univariate analyses, abdominal pain, peritoneal irritation, higher CRP, creatinine, and lactate, CT findings of ascites or reduced bowel wall enhancement, and linear PI pattern were associated with the presence of bowel necrosis. Postoperative patients with PI or PVG were more likely to have bowel necrosis than patients who had not had surgery (*P* = 0.003) (Table 3).

**Table 3:** Multivariate analysis of predictors of bowel ischemia

Variable	95 % CI	*P* value	Odds ratio
Postoperative detection (yes or no)	0.142–7.05	0.003	1.43
Deterioration in bowel wall enhancement (yes or no)	0.021–64.53	0.951	0.894
Abdominal pain (yes or no)	0.0029–5.00	0.234	0.1309
Peritoneal irritation signs (yes or no)	0.00083–17.14	0.213	0.554
Ascites (yes or no)	0.545–269	0.12	0.123
Linear pneumatosis intestinalis pattern (yes or no)	0.00183–2.79	0.207	0.137
Lactate (≥3 or < 3 mmol/L)	0.0892–14.95	0.68	1.96
C-reactive protein (≥3 or <3 mg/dl)	0.0232–16.62	0.89	0.806
Creatinine (≥1.2 or < 1.2 mg/dl)	0.0127–16.72	0.76	1.87

*CI* Confidence interval

### Characteristics of patients with postoperatively detected PI/PVG

Postoperative PI/PVG was detected in 9 (17.3 %) patients (Table 4). Eight had PI, 4 had PVG, and 3 had both. Eight of these patients had bowel necrosis; 5 died, and 3 were successfully treated with further surgical intervention. One patient developed PI/PVG and NOMI was suspected after aortic replacement for an abdominal aortic aneurysm; this individual was treated conservatively.

**Table 4:** Patients with postoperative pneumatosis intestinalis (PI)/portal venous gas (PVG)

Bowel necrosis	Sex	Age (y)	PI/PVG	Preoperative diagnosis	Initial surgery	Time to postoperative PI/PVG (days)	Postoperative diagnosis	Postoperative treatment	Outcome
Yes	Male	20	PI	Appendicitis	Appendectomy	7	Postoperative abscess	Open abdominal drainage	Alive
Yes	Female	84	PI/PVG	Sigmoid colon cancer	Sigmoidectomy	3	NOMI	Exploratory laparotomy	Dead
Yes	Male	67	PI/PVG	Perforated appendicitis	Intraperitoneal irrigation and drainage	1	Perforated appendicitis	Intraperitoneal irrigation & drainage	Dead
Yes	Female	48	PI	Metastases of uterine cancer, obstructive ileus	Radical hysterectomy + partial small bowel resection	24	Obstructive ileus	Observation + antibiotic therapy	Dead
Yes	Female	56	PI	Sigmoid colon cancer	Sigmoidectomy	3	Anastomotic breakdown	Hartmann’s operation + construction of artificial anus	Alive
Yes	Male	74	PI/PVG	Esophageal cancer	Subtotal esophagectomy	6	Toxic megacolon	Observation + antibiotic therapy	Dead
Yes	Female	55	PI	Uterine cancer	Total hysterectomy	16	Strangulated ileus	Ileus surgery + partial small bowel resection	Alive
Yes	Male	55	PVG	Pancreatic head cancer	Pancreaticoduodenectomy	15	Postoperative pancreatic fistula	Observation + antibiotic therapy	Dead
No	Male	82	PVG	AAA	Aortic replacement	1	NOMI suspicion	Observation + antibiotic therapy	Alive

*NOMI* non-occlusive mesenteric ischemia, *AAA* abdominal aortic aneury

## Discussion

PI is the presence of gas within the submucosa or subserosa of the intestine. A previous study found that the mean age of PI patients is 56.6 ± 19.4 years and that 57 % of patients were male [8]. PI is a rare disease and is still poorly understood. Four main theories exist, positing that PI is caused by: (1) increased intraluminal pressure derived from intestinal obstruction, colonoscopy, or gastroenteric tumor (mechanical theory); (2) pulmonary alveolar rupture resulting from pulmonary diseases, such as chronic obstructive pulmonary disease or asthma (pulmonary theory); (3) gas produced by gas-forming bacteria that enter the mucosal barrier (bacterial theory); or (4) α-glucosidase inhibitors (chemical theory) [9]. However, each theory alone also fails to account for the cause of PI [2, 3].

PVG is the presence of air in the portal venous system. The mechanism for the appearance of gas in the portal vein is also not well understood. There are two major theories: (1) gas dissects into the intestinal wall from the intestinal lumen, or lung and intestinal mucosal damage allows intraluminal gas to enter the venous system (Mechanical theory); and (2) gas-forming bacteria enter the mucosal barrier and produce gas within the intestinal wall, which then enters into the portal vein system (bacterial theory) [4, 10].

Previous reports have suggested that PI and PVG usually originate from bowel necrosis, via similar mechanisms, and are often detected concomitantly in clinical situations. However, PI and PVG have been studied and treated as independent symptoms in many publications. In the current report, we treated patients with PI and/or PVG as having one condition [5].

PI was previously thought to predict poor outcomes due to the development of bowel necrosis, but DuBose et al. evaluated 500 patients with PI and reported that it was not necessarily associated with bowel necrosis [8]. Similarly, PVG was also thought to be indicative of a poor prognosis due to the development of bowel necrosis. This is supported by the findings of Kinoshita et al., who reported that the overall mortality rate for patients with PVG was 39 %, increasing to 75 % in patients with bowel necrosis and only 11.7 % in patients without necrosis [4]. Furthermore, 97 % of PVG cases were reported to be related to bowel necrosis [11]. In recent years, the widespread use of CT has enabled increased detection of PI/PVG, showing that these clinical signs are not always predictive of a poor outcome. However, this is because bowel necrosis is not always found in some patients with PI and/or PVG. The increased sensitivity of CT scans has increased the detection of PI and PVG, increasing the difficulty of determining whether or not a patient with PI/PVG has bowel necrosis. Some authors have reported the development of therapeutic algorithms for treating PI/PVG [5, 12], but the wide variety of potential clinical features makes the development of an algorithm that is applicable to all patients with PI/PVG difficult.

Some clinical findings have also been suggested to predict bowel necrosis in patients with PI/PVG. Some reports suggested that bowel necrosis was associated with the distribution of PVG and bowel gas morphology, though conflicting reports have made this suggestion controversial [7, 13–15]. Bowel necrosis could not be predicted from the distribution or extent of PVG/PI. The distribution of PVG was entirely unrelated to bowel necrosis, whereas linear PI had a nonsignificant association with bowel necrosis. Patient characteristics and past medical histories have been reported to influence the occurrence of PI/PVG [15, 16], but we found no such relationship. Many laboratory tests have also been reported to be useful for the diagnosis of PI/PVG. Some reports have suggested that blood lactate levels and acidosis, indicators of tissue necrosis, are predictive of outcomes [8, 17, 18]. Our univariate analysis showed that elevated levels of lactate, C-reactive protein, and creatinine were significantly associated with bowel necrosis. These laboratory parameters are related to tissue necrosis, inflammation, and dehydration, suggesting that our findings are reasonable. Furthermore, abdominal physical examination findings are important predictors of bowel necrosis. In particular, signs of peritoneal irritation have been thought to be useful for diagnosing peritonitis due to bowel necrosis [19]. Our study supported this contention, suggesting that both abdominal pain and signs of peritoneal irritation predict bowel necrosis. However, the assessment of physical findings is physician-dependent, making an objective or numerical expression of such findings difficult. Several scoring systems that evaluate patient condition severity have been put forth as useful prognostic indicators for patients with bowel necrosis [20, 21]. In this study, the SIRS, APACHE II, and SOFA scores were used to assess severity. All three scores were related to bowel necrosis, indicating that these scores can be useful for evaluating patients undergoing surgery or conservative treatment. Recently, procalcitonin and presepsin may indicate useful prognostic information for patients with severe sepsis or septic shock. These parameters are expected to be useful for evaluating the severity of patients’ condition in the future [22].

PI/PVG is more likely to indicate bowel necrosis in postoperative patients than in patients who have not had surgery. There are several reasons that postoperative PI/PVG may be an indicator of bowel necrosis. First, the proliferation of intestinal bacteria and the production of gas in the bowel increase the intraluminal pressure, particularly in patients with postoperative ileus; this may lead to bowel ischemia and PI/PVG. Second, inflammatory cytokines increase vascular permeability, and the resulting intravascular dehydration may cause bowel ischemia [23]. In our study, the decrease in intravascular fluid, secondary to severe operative stress and perioperative catecholamine use, may have caused bowel ischemia in patients who underwent highly invasive surgeries, such as esophagectomies or pancreaticoduodenectomies. Third, the intestine can be damaged during abdominal surgery, and suture failure sometimes occurs after bowel resection and anastomosis. If there is massive leakage of bowel contents into the abdominal cavity, re-operation may be required due to bowel necrosis or peritonitis. Moreover, vasoconstrictors or vasopressors (e.g., catecholamines) are used if the patient’s blood pressure is unstable. Although vasoconstrictors increase blood pressure, they may also compromise blood flow to the peripheral intestinal vessels. Excessive vasoconstriction or repeated alternation of vasoconstriction and vasodilatation may also induce mesenteric artery spasms, causing ischemic enteritis or NOMI [24, 25]. Diagnosis of PI/PVG in postoperative patients is thus a simple indicator of potential bowel necrosis and may be useful for determining treatment.

In many cases, PI or PVG occurs in patients who have not undergone surgery, and it appears to resolve spontaneously in many of these patients. This is because PI or PVG can occur due to transient and reversible intestinal ischemia under certain conditions. For example, gas-forming bacteria can enter the mucosal barrier, through mucosal rents or increased mucosal permeability, and produce gas within the bowel wall; alternatively, the intestinal wall may be injured, or increased intraluminal pressure may serve as the driving force in PI that causes the intramural gas, due to conditions such as intestinal obstruction, inflammatory bowel disease, bowel preparation, or colonoscopy.

Our study had several limitations, including being a retrospective, single-center investigation involving a small sample size. The PI/PVG and bowel ischemia diagnoses were dependent upon the subjective judgment of the attending physicians, leading to potential selection bias. Furthermore, this study did not evaluate patients with bowel necrosis who did not have PI and/or PVG, so the association between PI/PVG and bowel necrosis cannot be fully assessed. The pathogenesis and clinical significance of PI/PVG should thus be further investigated in future multicenter prospective studies.

## Conclusion

In conclusion, postoperative detection of PI/PVG is indicative of bowel necrosis and is a useful indicator for surgeons deciding between further surgical intervention and conservative therapy.

## Abbreviations

APACHE II, Acute Physiology and Chronic Health Evaluation II; CRP, C-reactive protein; CT, computed tomography (CT); NOMI, non-occlusive mesenteric ischemia; PI, pneumatosis intestinalis; PVG, portal venous gas; SOFA, Sequential Organ Failure Assessment score; SIRS, Systemic Inflammatory Response Syndrome score
